# Antiperiodic Properties of the Humulus Lupulus

**Published:** 1853-11

**Authors:** W. Y. Gadberry

**Affiliations:** Benton, Miss.


### MATERIA MEDICA AND THERAPEUTICS.

*Antiperiodic Properties of the Humulus Lupulus.* By W. Y. Gadberry, M.D., of Benton, Miss.—As a substitute for quinine is a great desideratum on account of its enhanced market value, I have thought a brief notice of the antiperiodic virtues of the humulus lupulus, or common hop, might not be unacceptable to the profession. I am not aware that any author has ascribed to this plant any such virtue. Having used it for nearly two years I can confidently state that its antiperiodic properties equal, if they do not exceed, those of any other article of the materia medica with which we are acquainted, quinine alone exc pted; and, indeed, in my experience, it has often succeeded in arresting intermittents after that remedy had failed. It is harmless in its effects, and will often be borne by patients who cannot take quinine.

Every practitioner is aware of the advantage of combining an anodyne with antiperiodics; and by reference to the works on materia medica, the reader will see that hops possess these properties. When administered alone the infusion is preferable, and should be made of double the strength prescribed by the Dispensatory. One ounce infused in a pint of boiling water may be taken during the interval, or a larger quantity if necessary. If the secretions are properly regulated, and there exists no enlargement of the spleen, it will rarely fail to effect a cure of tertian or quartan ague. It has not succeeded so well in the cases of quotidian type as in those of more protracted intervals. The tincture was used alone in three cases successfully. The following combination is worthy a trial by all who desire a safe and efficient substitute for quinine:

℞. Tinct. hops, tinct. Peruvian bark, aa ℥ iv.

Pulv. black pepper, ℥ ss.

To be given in doses of half an ounce every two hours during the interval.

My limited experience will not justify an opinion upon the antiperiodic virtue of lupuline, not having used it except in combination of quinine. Patients to whom I have administered this combination prefer it to quinine alone, on account of its soothing effect upon the nervous system. The hop is indigenous to this country, growing abundantly in almost every garden; and if I have not over estimated its antiperiodic virtue, it will prove a blessing to the poor, in whose welfare the physician should always feel a special interest.—*West. Journ. Med. and Surg.*

